# Synchronous Lobular Breast Cancer and Lung Adenocarcinoma: Α Case Report

**DOI:** 10.7759/cureus.109435

**Published:** 2026-05-22

**Authors:** Varvara Pantelidou, Evangelos Perdikakis, Eleftherios Eleftheriadis, Savvas Lampridis, Ioanna Tsantila, Athanasios Permekerlis

**Affiliations:** 1 Surgical Department, 424 General Military Training Hospital, Thessaloniki, GRC; 2 Interventional Radiology Department, 424 General Military Training Hospital, Thessaloniki, GRC; 3 Pathology Department, Istodierevnitiki SA, Thessaloniki, GRC; 4 Cardiothoracic Surgery Department, 424 General Military Training Hospital, Thessaloniki, GRC; 5 Faculty of Medicine, Imperial College London, London, GBR; 6 Pathology Department, 424 General Military Training Hospital, Thessaloniki, GRC

**Keywords:** immunohistochemistry, lobular breast cancer, lung adenocarcinoma, mastectomy, multidisciplinary team, thoracotomy

## Abstract

Synchronous breast and lung cancer (SBLC) is a rare entity posing great challenges in diagnosis and treatment. A 58-year-old female was referred to the breast department of our hospital with a palpable mass in the right breast. Mammography, ultrasound, and magnetic resonance (MR) imaging revealed bilateral, multifocal breast lesions. Core needle biopsy (CNB) revealed pleomorphic lobular breast cancer in the right breast and ductal carcinoma in situ (DCIS) in the left breast. Staging positron emission tomography-computed tomography (PET-CT) showed a suspicious lesion in the left lung. CT-guided biopsy revealed lung adenocarcinoma. According to the multidisciplinary team (MDT) decision, the patient underwent a staged approach. First, she was scheduled for bilateral mastectomy and right sentinel lymph node biopsy (SLNB). One month later, she underwent a left upper lobectomy and lymphadenectomy through a left posterior mini-thoracotomy. At present, two years later, the patient is currently in remission. Thus far, there are no clear and unified treatment guidelines for SBLC. Therefore, such cases should be discussed in MDT meetings, taking into account the stage of each cancer, the morbidity and mortality of each therapeutic approach, together with the patient’s comorbidity and preference.

## Introduction

Multiple primary malignant tumors (MPMTs) are defined when two or more primary malignant tumors are present in different organs in the same patient [1]. The diagnostic criteria of MPMTs, first described by Warren and Gates, are the following: (a) there should be histological confirmation of malignancy in each of the tumors, and (b) each tumor must be geographically separate and distinct. The lesions should be separated by normal mucosa; (c) the probability of one being the metastasis of the other must be excluded. According to international rules for multiple primary cancers, if multiple cancers occur in the same organ or paired organs of the same tissue origin, they are considered multifocal cancers [2]. Among cancer patients, the incidence of this uncommon entity varies from 0.73% to 11.7% and nowadays is increasing due to advancements in imaging techniques [1].

The lung is the most common metastatic site of breast cancer [3]. Differential diagnosis of pulmonary nodules detected in staging thoracic computed tomography (CT) includes primary lung cancer (50%), metastatic lesions (37%), and benign lesions (8%). CT-guided biopsy, pathologic examination, and immunohistochemistry testing are of paramount importance for diagnosis and treatment planning [4,5].

Herein, we present a rare case of a 58-year-old female with synchronous lobular breast and lung adenocarcinoma.

## Case presentation

A 58-year-old post-menopausal female was referred to the Breast Department of our hospital with a palpable mass in the right subareolar area. She had a history of atrial fibrillation, ischemic stroke, obesity, Hashimoto thyroiditis, and depression. She was a heavy smoker with 30 pack years and had a body mass index (BMI) of 42.5. Mammography revealed abnormal findings in the upper quadrants and in the periareolar area of both breasts, and an ultrasound was performed (Figure 1). Ultrasound depicted an irregular hypoechoic lesion on the right breast, with dimensions 12 × 8.5 × 8.5 mm, stiff and heterogeneous in elastography, classified as Breast Imaging Reporting and Data System (BI-RADS) 5 (Figure 2). The axillary lymph nodes were considered benign and hyperplastic sonographically. Breast magnetic resonance imaging showed an irregular solid nodular lesion in the right retroareolar area, with dimensions 17 × 12 × 18 mm, with intense contrast enhancement (enhancement curve type III), a satellite nodule with a similar enhancement pattern, with dimensions 6 × 5 mm, at 12 o’clock, and also small multiple foci of enhancement. There was also a suspicious lesion in the left breast, at 5 o’clock, with a diameter of 8 mm, with lobular irregular margins and enhancement pattern type II (Figure 3).

**Figure 1:**
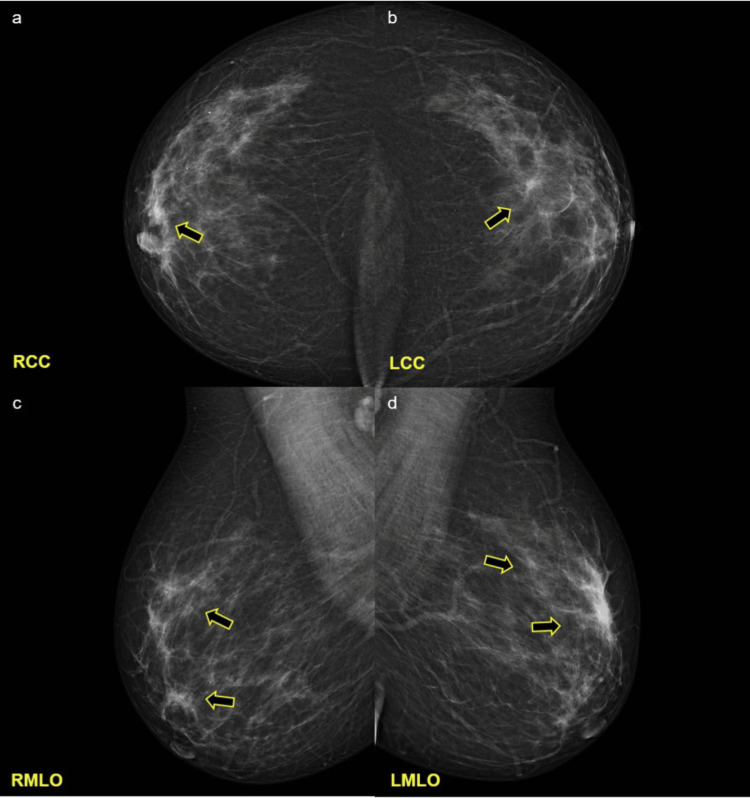
Mammographic examination The craniocaudal (a-b) and lateral oblique (c-d) mammographic images demonstrate abnormal findings in the upper quadrants and in the periareolar area of both breasts (arrows). The findings were studied with supplementary ultrasound.

**Figure 2:**
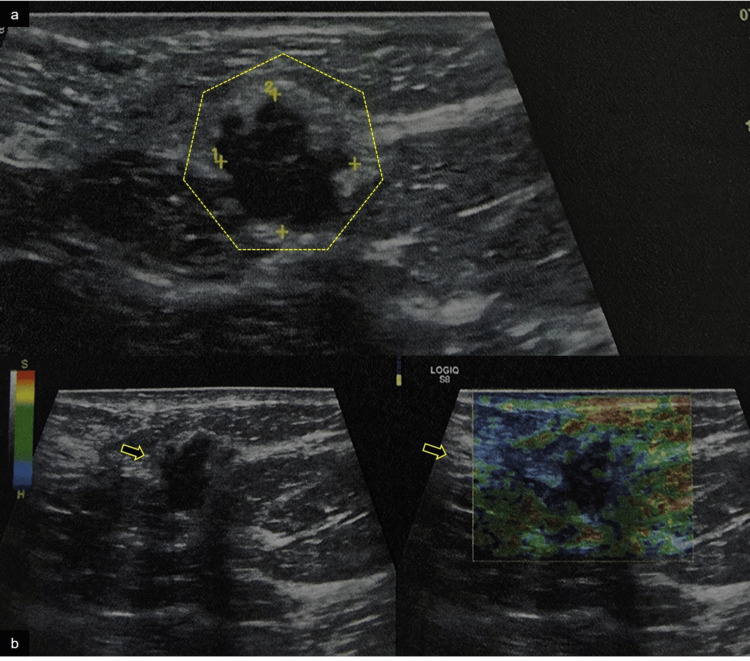
Ultrasound examination The ultrasound examination (a) shows tumor on the right breast (yellow dotted area) and the additional elastographic images (b) demonstrate decreased elasticity of the breast tumor with tissue strain imaging.

**Figure 3:**
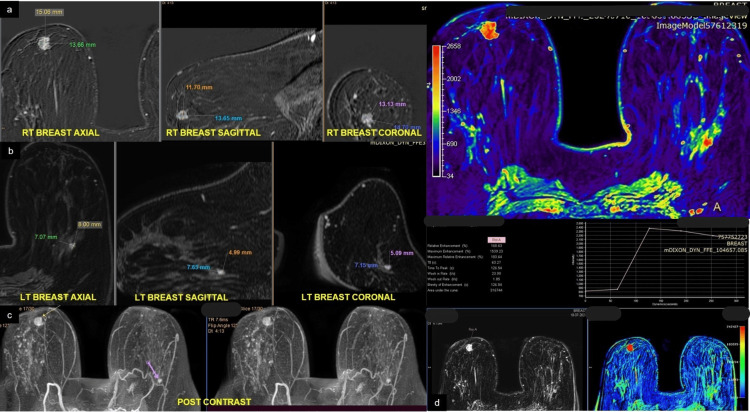
Breast magnetic resonance imaging (a-b) The digital subtraction T1-weighted contrast-enhanced MRI images show abnormal intense uptake on the right and left breast. (c) The digital subtraction T1-weighted contrast-enhanced MRI MIP reconstructed images show the abnormal nodular uptake on both breasts. (d) Intense contrast enhancement (enhancement type III) of the main lesion on the right breast.

A core needle biopsy (CNB) of both lesions was performed. The right breast was a pleomorphic lobular carcinoma grade 2. Immunohistochemistry testing revealed negativity in p63 and positivity in E-cadherin immunostaining. The left breast was ductal carcinoma in situ (DCIS) grade 1. The patient underwent a thoracic and abdominal computed tomography (CT) scan, which revealed an irregular hyperdense area, with dimensions 15 × 21 mm in the apical-posterior segment of the upper lobe of the left lung. Afterward, she had a positron emission tomography-computed tomography (PET-CT) scan that demonstrated a hypermetabolic lesion in the apical-posterior segment of the upper lobe of the left lung (SUVmax = 4.6), consistent with malignancy, and was submitted to CT-guided biopsy (Figure 4). No other evidence of metastatic disease. Immunohistochemistry testing revealed estrogen receptor (ER) and GATA binding protein 3 (GATA-3) negativity and positivity in CK-7, Napsin A, and thyroid transcription factor-1 (TTF-1) immunostaining. Pathology and immunohistochemistry were indicative of lung adenocarcinoma acinar, solid, and lepidic type (Figure 5).

**Figure 4:**
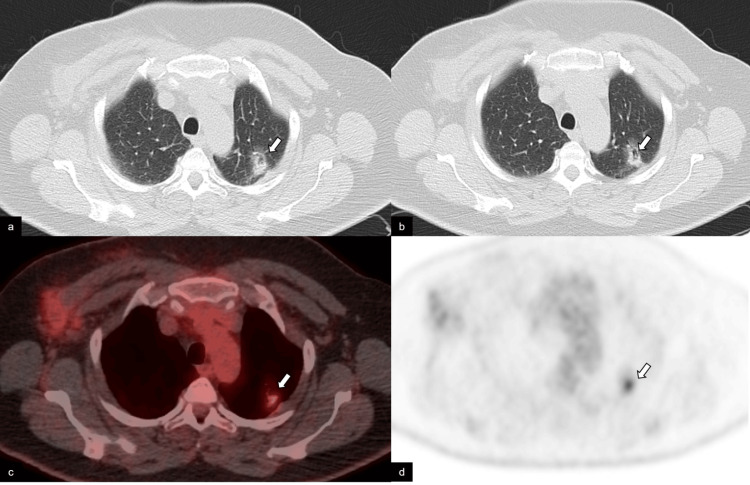
Positron emission tomography-computed tomography (PET-CT) The axial (a-b) CT images and the corresponding (c-d) PET-CT images show an irregular hyperdense area, in the apical-posterior segment of the upper lobe of the left lung (arrows) that demonstrated increased uptake of 18F-FDG (SUVmax =4.6). Mediastinal pool SUV = 2.6. SUVmax: maximum standardized uptake value; SUV: standardized uptake value; 18F-FDG: 18F-fluorodeoxyglucose

**Figure 5:**
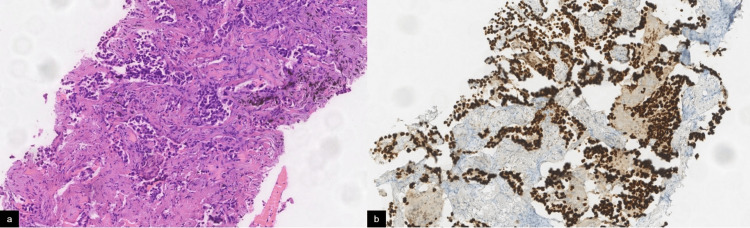
Histopathologic examination of CT-guided lung tumor specimen (a) Hematoxylin-eosin stain, magnification view x10, show acinar, solid, and lepidic-type neoplastic cells. (b) ΤΤF-1-positive immunohistochemistry stain, magnification view x10, indicative of lung adenocarcinoma.

The case was discussed in the multidisciplinary team (MDT) meeting, and the surgical treatment of both cancers was decided to take place in two stages: breast cancer surgery followed by lung cancer surgery. After discussion with the patient, she preferred a bilateral mastectomy, because she did not want to receive radiotherapy. Therefore, she was scheduled for bilateral mastectomy and right sentinel lymph node biopsy (SLNB). Three sentinel lymph nodes were removed, which were free of disease, both in the frozen section and the final specimen. Final histopathologic results were as follows. Right breast: Invasive lobular carcinoma, mainly pleomorphic and in a smaller percentage, solid and classic variant, diameter 2.2 cm, grade 2, pT2(is)N0. Immunohistochemistry testing revealed estrogen receptor (ER) positivity (H-score 230), progesterone receptor (PR) positivity (H-score 180), human epidermal growth factor receptor (Her-2) negativity, moderate p53 positivity, and E-cadherin aberrant positivity, exhibiting cytoplasmic, fragmented, or weak membranous staining and only focal strong membranous staining. The stain for β-catenin was negative throughout the neoplastic cells. Multiple foci of DCIS, grade 1 and 2. Few foci of lobular carcinoma in situ (LCIS). Of the tumor cells, 18% were positive for the Ki-67 immunostain. The left breast was DCIS grade 1, with a diameter of 5 mm, stage IIA (Figures 6-7).

**Figure 6:**
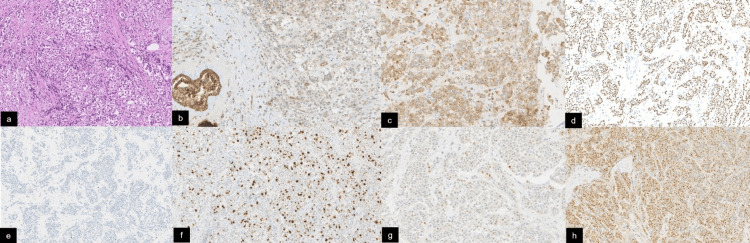
Histopathologic examinations tumor of the right breast (a) Hematoxylin-eosin stain, magnification view x10. (b) Catenin-negative immunohistochemistry stain, magnification view x10. (c) Cadherin-positive immunohistochemistry stain, magnification view x10. (d) ER strong positive, magnification view x10. (e) HER2-negative immunohistochemistry stain, magnification view x10. (f) 18% of the tumor cells were positive for the Ki-67 immunostain, magnification view x10. (g) P53 moderately positive immunohistochemistry stain, magnification view x10. (h) PR-positive immunohistochemistry stain, magnification view x10. Immunohistopathologic findings compatible with pleomorphic lobular breast cancer.

**Figure 7:**
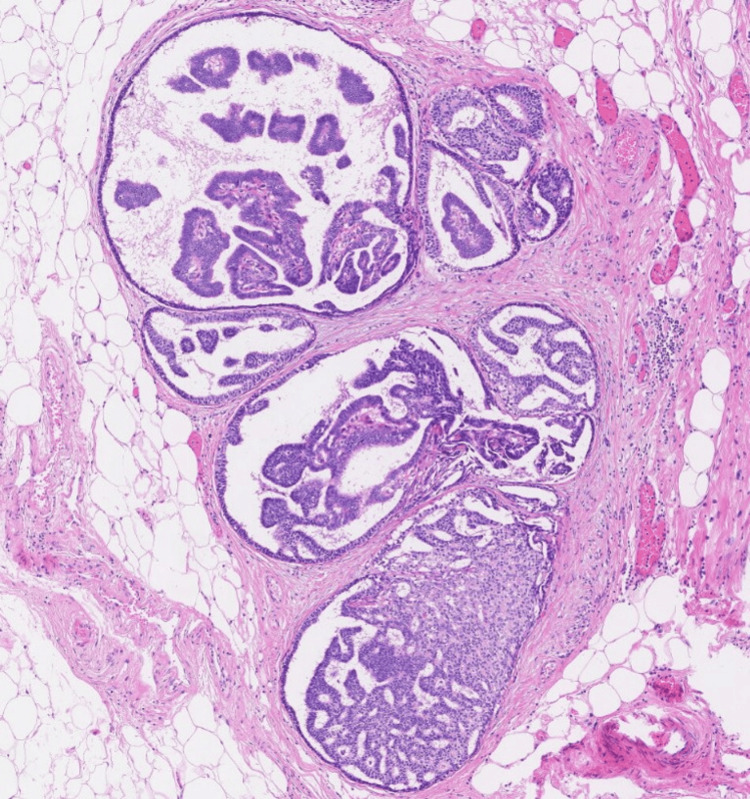
Histopathologic examination tumor of the left breast Hematoxylin-eosin stain, magnification view x10, shows ductal carcinoma in situ (DCIS) grade 1.

One month later, the patient underwent left upper lobectomy and lymphadenectomy of lymph node stations 5 and 10 L through a left posterior mini-thoracotomy. The final histopathologic exam revealed invasive non-mucinous adenocarcinoma, acinar type, grade 2, max diameter 1.4 cm, T1bN0. Immunohistochemistry testing revealed negativity in GATA-3, P40, ER, CD 56, and P63 and positivity in CK7, TTF-1, and CK8/18 immunostaining (stage IA2) (Figure 8). Pathological findings of breast and lung cancer are summarized in Tables 1-2.

**Figure 8:**
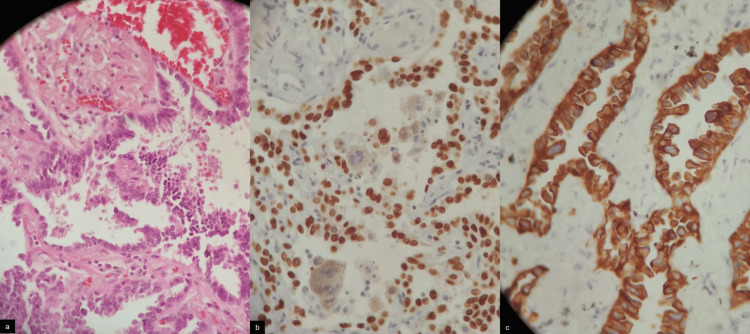
Histopathologic examination of lung lobectomy Histopathologic examination of lung lobectomy revealed an invasive non-mucinous adenocarcinoma, acinar-type grade 2. (a) Hematoxylin-eosin stain, magnification view x400: Invasive non mucinous lung adenocarcinoma (WHO 2022-AJCC 8th edition). (b) Immunohistochemistry TTF-1 stain, magnification view x400. Lung adenocarcinoma with strong nuclear positivity in cancer cells. (c) Immunohistochemistry CK8/18 stain, magnification view x400. Lung adenocarcinoma with strong membranous positivity in cancer cells.

**Table 1:** Pathological findings of breast cancer DCIS: ductal carcinoma in situ, ER: estrogen receptor, PR: progesterone receptor, Her-2: human epidermal growth factor receptor 2

Pathological findings	Breast cancer
Histological subtype	Invasive lobular pleomorphic, multiple foci of DCIS
Tumor size	2.2 cm
TNM stage	pT2(is)N0 / stage IIA
Ki-67	18%
Immunohistochemical markers	ER, PR, p53, E-cadherin, catenin, Her-2
Receptor status	ER, PR, p53, and E-cadherin positive/catenin and Her-2 negative

**Table 2:** Pathological findings of lung cancer GATA-3: GATA binding protein 3, ER: estrogen receptor, CK: cytokeratin, TTF-1: thyroid transcription factor

Pathological findings	Lung cancer
Histological subtype	Invasive non-mucinous adenocarcinoma acinar type
Tumor size	1.4 cm
TNM stage	pT1bN0/ stage IA2
Immunohistochemical markers	GATA-3 and ER negative/CK7, TTF-1 and CK8/18 positive

Postoperatively, the patient did not receive any adjuvant therapy. She is followed up every six months with clinical history, physical examination, and thoracoabdominal CT. During the two-year follow-up, the patient did not show any recurrence of breast or lung cancer or any complication of bilateral mastectomy or thoracotomy. She continues hormone therapy (letrozole 2.5 mg 1 x 1) according to the guidelines.

## Discussion

MPMTs are further classified as synchronous, when the time interval between the diagnosis of the first and second cancer is less than six months, and metachronous, when the time interval is more than six months [6]. According to the Surveillance, Epidemiology, and End Results programme, synchronous primary cancers are defined as those diagnosed within two months interval. Based on the aforementioned data, in 1.7% of breast cancer patients, another synchronous cancer is detected [7]. As far as SBLC is concerned, the reported incidence is even lower. A recent retrospective study, among 8,204 patients with breast cancer, depicted four patients with a synchronous lung cancer (<0.05%) [8]. In another retrospective study with 1,066 patients, six were diagnosed with SBLC [3].

In breast cancer patients, the distinction between synchronous primary lung adenocarcinoma and lung metastasis, based only on CT imaging features, is difficult. Therefore, tissue biopsy and immunohistochemistry in suspected metastatic lesions are of paramount importance [8]. Thyroid transcription factor 1 (TTF-1) and napsin A are two markers, usually used to distinguish primary from metastatic lung adenocarcinoma. According to a tissue microarray study, positivity of both aforementioned immunostains provides a good sensitivity (84.9%) and an excellent sensitivity to distinguish primary lung adenocarcinoma [9]. The presented case fulfills the diagnostic criteria of synchronous MPMTs, since primary lung cancer was first detected in the thoracic CT scan, required for staging breast cancer. In our case, preoperative immunohistochemistry testing of the lung lesion revealed negativity in GATA-3 and ER, excluding the possibility of breast cancer metastasis. Positivity in CK-7, Napsin A, and TTF-1 immunostaining was indicative of lung adenocarcinoma.

However, in cases of triple-negative breast cancers, negativity of suspected lung lesions in ER, PR, and HER-2 immunostains cannot completely exclude the possibility of metastatic breast cancer. Moreover, negative expression of TTF-1 and napsin A cannot completely exclude the possibility of primary lung adenocarcinoma. In such equivocal cases, next-generation sequencing (NGS) can distinguish primary from metastatic lesions, revealing whether breast and lung lesions derive from the same clone [8].

Prompt diagnosis and treatment of a synchronous lung adenocarcinoma in patients with BC is important for two reasons. First, patients with SBLC have higher hazards of death compared to patients with BC only [10]. Second, prompt diagnosis of asymptomatic incidentally detected lung cancer has more chances of treatment with radical lung surgery, which significantly affects prognosis. By contrast, failure to undergo radical lung cancer surgery is an independent risk factor for progression-free survival (PFS) and overall survival (OS). Double cancer resection reassures a better prognosis [11].

As far as histology is concerned, the majority of SBLC cancers are invasive ductal breast carcinomas with lung adenocarcinoma [8]. In our case, breast cancers were invasive pleomorphic lobular carcinoma in the right breast and in situ ductal carcinoma in the left breast. Another point that deserves mentioning in this case is that the lobular carcinoma was e-cadherin positive. Invasive lobular cancer (ILC) accounts for 10-15 % of invasive breast tumors and is characterized by inactivation of E-cadherin. E-cadherin, encoded by the CDH1 gene on chromosome 16q22.1, is a transmembrane glycoprotein essential for epithelial cell adhesion. The hallmark molecular alteration in ILC is a loss-of-function mutation in CDH1, which encodes the adhesion protein E-cadherin. Inactivation of E-cadherin leads to the inability of tumor cells to adhere to one another and is responsible for the characteristic single-file infiltration pattern and discohesive morphology of ILC. However, approximately 5-10 % of ILCs retain aberrant E-cadherin expression. On the other hand, loss of E-cadherin does not exclude ILC. Therefore, immunostaining should be interpreted in conjunction with morphology to avoid misclassification [12].

Although not rare, ILC is not adequately represented in research compared to invasive ductal carcinomas (IDCs). Therefore, detection, treatment, and follow-up strategies are based on data from IDC. However, ILC presents unique features in terms of detection, histology, and clinical manifestation. It is characterized by multifocality and multicentricity at presentation, and its unique pathology results in underestimation of the extent of the disease in preoperative imaging. Therefore, more extensive surgery is required to obtain clear margins, leading to higher rates of mastectomy compared to IDC [13]. In our case, breast MRI revealed multifocal disease and played an important role in the final decision of bilateral mastectomy.

In general, ILC has a worse prognosis than IDC [13]. In our case, the right breast cancer consisted mainly of a pleomorphic variant of ILC, which usually has a higher mitotic rate and worse prognosis compared to the classic type. Consequently, although the final pathologic stage of breast cancer is T2N0, pleomorphic variant of ILC, multifocality, and the presence of DCIS in both breasts are indicative of an extensive breast disease, which requires prompt intervention.

Currently, no clear and unified clinical treatment guidelines for synchronous primary cancers have been developed [3]. They are commonly reported as case reports lacking systemic analysis. Treatment strategy for synchronous breast and lung cancer (SBLC) is challenging due to a lack of specific screening and well-established guidelines. Further research is needed to increase our understanding, while MDT is required [8]. Stepwise approach takes into account the morbidity and mortality of each cancer type and its treatment, combined with the patient’s comorbidity and preference. After communication with the patient, she decided on a bilateral mastectomy because she wanted to avoid postoperative radiotherapy. The MDT meeting decided on a staged approach due to comorbidities, which would lead to severe postoperative morbidity if bilateral mastectomy and left thoracotomy were performed at the same time. Ιn addition to this, she was a heavy smoker, and she was recommended to stop smoking one month before thoracotomy. According to the National Comprehensive Cancer Network, active smokers present an increased incidence of postoperative pulmonary complications and therefore smoking cessation support should be offered [14].

## Conclusions

SBLC is a rare entity with great challenges in diagnosis and treatment. The therapeutic approach should take into consideration not only the disease stage, but also the patient’s comorbidities and the potential complications of each treatment. MDT meeting, including breast surgeon, oncologist, radiation oncologist, radiologist, pathologist, and thoracic surgeon, is of paramount importance in order to provide a maximum survival benefit for patients with the least possible complications and side effects.
